# Supplementary material for: Effect of elevated temperature on membrane lipid saturation in Antarctic notothenioid fish
Source: PeerJ. 2018 May 18;6:e4765. doi: 10.7717/peerj.4765 (PMC5961637; doi:10.7717/peerj.4765)
Supplement: Supplemental Information 2 [file peerj-06-4765-s002.docx]

# Fish harvest and husbandry

## Collection of TB and PB fish samples for the thermal acclimation experiment

Fish were primarily caught within McMurdo Sound, Antarctica, or the wider Ross Sea area. Fish collection was carried out during the austral summer of 2007 (October-November). Fish were caught using small fishing rods, baited with either small fish pieces or soft bait on barbless hooks. Typically at the fishing sites, 25 cm diameter holes were drilled through the sea ice (which ranged from 2-8 metres thick) using a motorised jiffy drill and fish were accessed through these holes. Alternatively, a hole-melter was used to create a 1 metre wide hole in the sea ice through which fish were accessed, sometimes with a heated wannagin over top. Holes were kept clear of sea ice by regular use of small dip-nets. Individuals of the benthic species, *T. bernacchii*, were caught in inshore waters at a water depth of 15-40 m. Individuals of the pelagic and schooling species, *P. borchgrevinki,* were collected from deeper water locations (up to 300m deep), near the ice runway in McMurdo Sound with sea ice cover less than 4m thick (typically 2 m) and away from seal activity. Following capture, and quick and careful hook removal, fish were immediately placed in an insulated container of seawater and transported to laboratory facilities at Scott Base, where they were held in flow–through aquaria with sea water temperature maintained at -1.0 ± 0.3 °C. Fish were held post-catching for a minimum period of three days to allow them to recover from handling stress. During the recovery period, fish were not fed and were maintained in a 24 hour light regime to replicate the austral summer conditions of Antarctica. Following the recovery period, and when sufficient fish had been caught for the experiment, fish were pre-acclimated to a temperature of -1 °C for a period of 15 days and were fed *ad libitum* twice weekly.
